# Lens-induced myopization and body weight in young guinea pigs

**DOI:** 10.1186/s12886-023-03271-y

**Published:** 2024-01-03

**Authors:** Hao-Tian Wu, Xu-Han Shi, Li Dong, Rui-Heng Zhang, Yi-Tong Li, Wen-Bin Wei

**Affiliations:** Beijing Tongren Eye Center, Beijing Key Laboratory of Intraocular Tumor Diagnosis and Treatment, Beijing Ophthalmology & Visual Sciences Key Lab, Medical Artificial Intelligence Research and Verification Key Laboratory of the Ministry of Industry and Information Technology, Beijing Tongren Hospital, Capital Medical University, 1 Dong Jiao Min Lane, Beijing, 100730 China

**Keywords:** Axial length, Body weight, Lens-induced myopia

## Abstract

**Background:**

To investigate the relationship between body weight and Axial length in guinea pigs.

**Methods:**

Forty pigmented guinea pigs were randomly divided into two groups, namely control group and negative lens-induced myopization (LIM) group. After measuring the baseline axial length and body weight (BW), guinea pigs of LIM group received bilateral negative lens-induced myopization using − 10.0 diopters lenses. One week later, the lenses were removed and biometric and ophthalmoscopic examinations were repeated.

**Results:**

Two groups of guinea pigs showed no statistical difference in initial body weight and eye axis length. Compared to the control group, the lens-induced group had a lower weight (*P* = 0.02) and a longer axial length (*P* < 0.01) at the end of study Neither at baseline nor at week 1 did AL correlate with BW in both groups (Control Baseline: *r* = 0.306, *P* = 0.19; Control Week1: *r* = 0.333, *P* = 0.15; LIM Baseline: *r*=-0.142, *P* = 0.55; LIM Week 1: *r* = 0.189, *P* = 0.42). Lens-induction had a significant effect on axial elongation (*P* < 0.01) while body weight had no impact on such aspect (*P* > 0.05).

**Conclusion:**

In guinea pigs of the same age, axial length was not correlated with body weight. Also, baseline body weight had no impact on natural axial length growth or lens-induced myopia. Lens-induction caused a significant reduction in body weight gain.

## Introduction

The lens-induced myopia model of guinea pigs is one of the most studied myopia models in laboratory simulations. In such a model, axial length (AL) is considered as one of the major biological indicators of the degree of myopia. However, it is well known that axial length prolongs with individual growth and development early in life, and the development of each individual differs from one another to a certain extent. Previous myopia experiments in guinea pigs also showed significant differences in eye axis between guinea pigs of the same age [1, 2]. Therefore, it is worth investigating whether these guinea pigs of the same age are at different stages of growth and development due to dietary and environmental factors and whether it is necessary to calibrate this possible bias when interpreting experimental results.

Body weight/Length is one of the important indicators of individual growth and development. Multiple studies have reported a significant association between body weight/height and axial length in children [3–5]. However, observed association between body weight and the onset of myopia might be confounded by lifestyle difference or environment exposures, such as the sedentary lifestyle. Therefore, in order to confirm the authenticity of this finding, it is important to be able to reproduce it in a laboratory environment.

Since guinea pigs have no tails, we were unable to standardize the length of the subjects, as was done by Chakraborty et al. on mice [6]. Therefore, we chose body weight as a single indicator of its growth and development. The aim of this study was to investigate the relationship between growth and development in guinea pigs, as represented by body weight, and growth in axial length under natural and lens-induced conditions.

## Methods

### Animals and ethics

The experimental study included 40 male pigmented guinea pigs (Cavia porcellus) aged 2–3 weeks at baseline. The study was approved by the Ethics Committee of Beijing Tongren Hospital, and the ARVO Statement and ARRIVE Guidelines for the Use of Animals in Ophthalmic and Vision Research were followed. Animals were obtained from the Fang Yuan Farm in Beijing, China.

### Study design

**Fig. 1:**
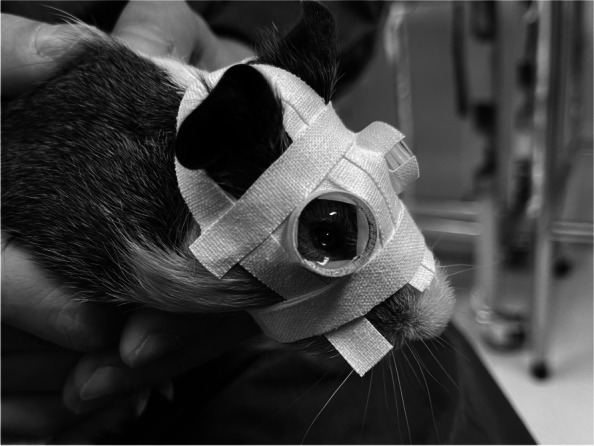
Guinea pig with bilateral lens induction

The animals were randomly divided into two groups and housed in 50*40*30 cm cages at a density of three per cage. Guinea pigs with the same treatment share the same cage. After measuring the baseline axial length and body weight of the two groups, the guinea pigs in LIM group received bilateral negative lens-induced myopization. As previously described in detail [7–9], goggles with a refractive power of -10.0 diopters were glued to the orbital rims of both eyes (Fig. 1), allowing the animals to open and blink freely. The animals were examined daily to ensure that the goggles were clean and in place. One week later, the goggles were removed and the axial length and body weight of both groups were repeated. At the end of the experiment, all animals were sacrificed by intraperitoneal injection of an overdose of pentobarbital sodium and their eyeballs were removed to be used as control material for other experiments.

### Axial length and body weight measuring

The Axial length of the right eye of each subject was measured by ocular ultrasound biometry (A/B mode scan; oscillator frequency: 11 MHz; Quantel Co., Les Ulis, France) under topical anesthesia (Proparacaine hydrochloride eye drops; Alcon NV, Belgium). Ten measurements were performed, and the mean values were used for further statistical analysis if the standard deviations of the measurements were less than 0.1 mm.

Body weight of each guinea pig was measured by an electronic scale (Accuracy 0.01 g, experimental data rounded to one decimal place). Three measurements were performed, and the mean values were used for further statistical analysis if the standard deviations of the measurements were less than 1 g.

### Statistics

For statistical analysis, we used a commercially available statistical analysis program (SPSS, version 26.0, IBM-SPSS, Chicago, IL, USA). Baseline axial length and body weight of the two groups were compared using two independent-sample T-test. The relationship between AL and BW at baseline and week 1 were analyzed by Pearson’s correlation analyses. The effects of body weight and lens-induction on the elongation of the axial length were analyzed using univariate analysis of variance (UNIANOVA). A *P*-value of less than 0.05 was considered to be statistically significant.

## Results

Baseline AL and BW of the two groups are shown in Table 1. Two groups showed no statistical difference in both baseline axial length and body weight.

**Table 1:** Axial length and Body weight at baseline and week 1

	Axial length (mm)		Body weight (g)	
	Baseline	Week 1	Baseline	Week 1
Control group (*n* = 20)	7.95 ± 0.06	8.06 ± 0.07	140.5 ± 9.85	198.6 ± 7.38
LIM group (*n* = 20)	7.95 ± 0.06	8.16 ± 0.05	142.9 ± 9.95	192.1 ± 9.14
*P*	0.85	< 0.001	0.45	0.02

Data was expressed as mean ± standard deviation

### Relationship between AL and BW

**Fig. 2:**
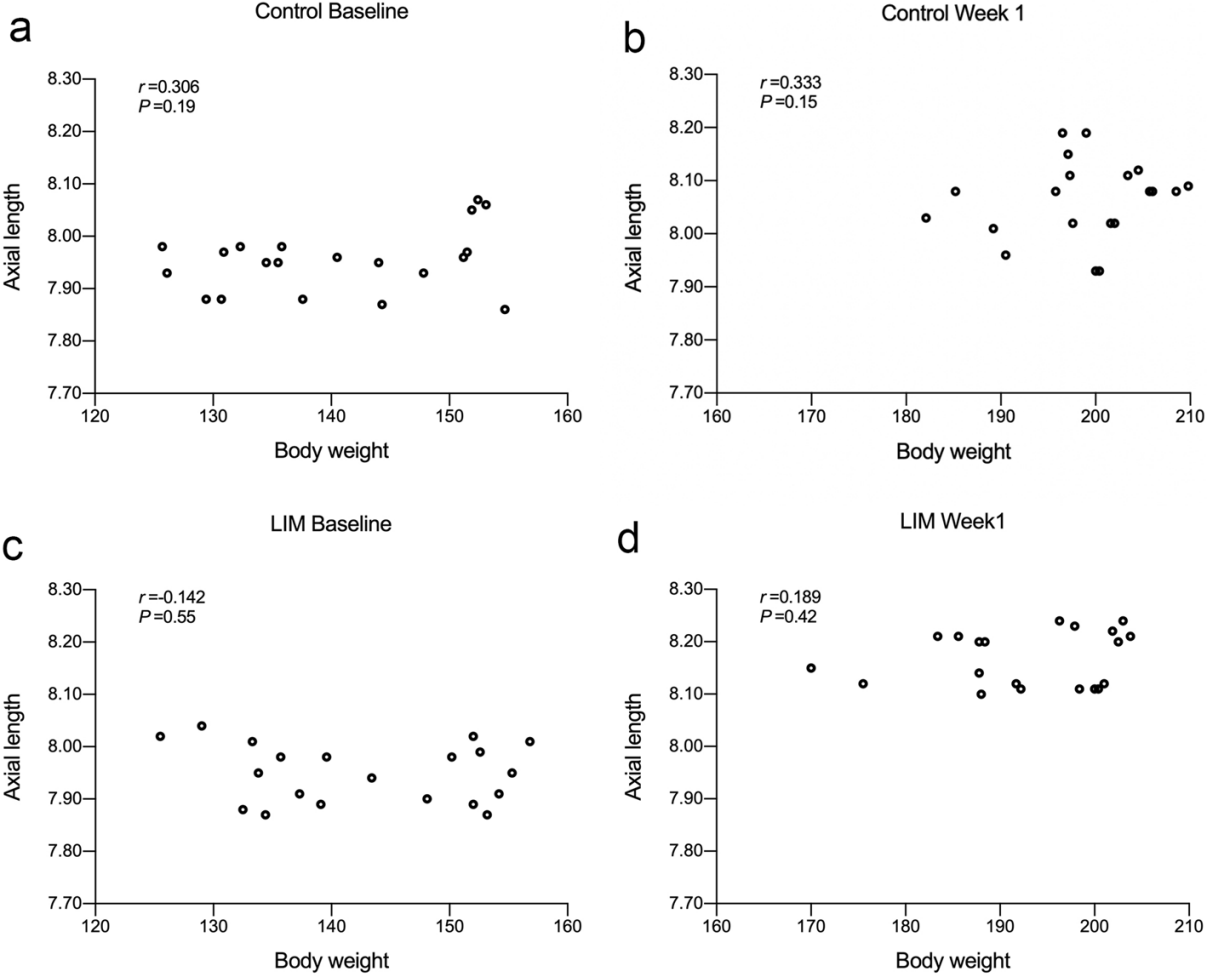
Correlations between AL and BW at baseline and week 1. **a** Correlation between AL and BW of control group at baseline. **b** Correlation between AL and BW of control group at week 1. **c** Correlation between AL and BW of LIM group at baseline. **d** Correlation between AL and BW of LIM group at week 1

Neither at baseline nor at week 1 did AL correlate with BW (Fig. 2).

### The effects of body weight and lens-induction on the elongation of the axial length

**Fig. 3:**
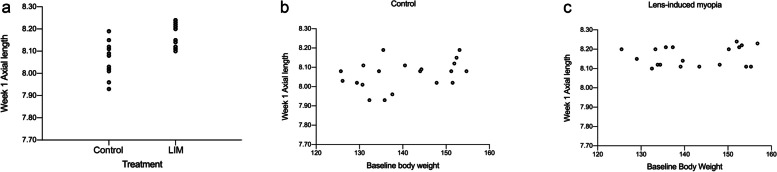
Effects of body weight and lens-induction on axial length. **a** Axial length in control group and LIM group at week 1. **b**, **c** Relationship between baseline body weight and week 1 axial length in control and LIM groups

Lens-induction had a significant effect on axial elongation (*P* < 0.01), Body weight had no impact on axial length (*P* > 0.05, Fig. 3).

## Discussion

There were complicated associations between myopia and body weight. Studies have shown that heavier children/adults tend to have longer AL, deeper vitreous chamber and flatter cornea than lighter persons of similar age and same sex [10]. However, they turn out to be more hyperopic. This phenomenon may be due to a flatter cornea resulting in a lower refraction, which compensates to some extent for the myopic changes caused by a longer axial length of the eye.

We demonstrated for the first time that, in guinea pigs of the same age, axial length was not correlated with body weight. Also, baseline body weight had no impact on natural axial length growth or lens-induced myopia. This finding not only addressed the concern in many animal studies that there may be a correlation between body growth and axial length development in guinea pigs, but also suggests to some extent that eye growth is closely related to chronological age rather than general body development. It also supports the rationale for using age as the basis for grouping in many myopia-related clinical trials.

In addition, this study also suggests that the observed association between body weight and the onset of myopia might be confounded by some factors, such as a sedentary lifestyle. For example, greater weight may reflect certain lifestyle choices, such as less outdoor activity, and less outdoor activity has been shown to be significantly associated with axial growth [11, 12]. Therefore, long axial length, along with heavier weight, may only be the common result of such factors.

Finally, it is worth noting that during this study we found that the mean body weight of the lens-induced myopia group was significantly lower than that of the control group at the end of the experiment. However, without further testing, we were unable to determine whether the lens induction simply affected body weight gain in some way (e.g. by affecting the eating behavior of the guinea pigs) or whether the overall development of the guinea pigs in the lens induction group was slower than that of the control group. This phenomenon has never been reported before and deserves further investigation in future experiments.

Although this study provided important result of lens-induced myopization and body weight in young guinea pigs, it has some limitations. First of all, since the body length of the subject could not be accurately determined as previous study done in mice [6], we were unable to use BMI (body mass index) as a more accurate indicator of the body size of guinea pig. Secondly, due to the lack of reliability of currently available refractive index measurements, we did not perform refractive index measurements on our subjects. For reference, Howlett et al. suggests that for guinea pigs of the same age as those used in the present study, it is possible to convert on a scale of 0.1 mm=-6D [13]. Finally, the observation of the axial elongation was not prolonged, therefore, whether body weight effects the long-term outcome of guinea pigs could not be determined. However, according to Howlett & McFadden [13], guinea pigs reach sexual maturity at only 75 days, which is equivalent to a 12 year old human (an approximate age ratio of 20:1). In other words, our short one-week experiment is equivalent to 6 months (or more) of human myopia development for a 4- to 6-year-old human. Thus, the length of the present study, although not entirely satisfactory for the study of juvenile myopia progression, is sufficiently illustrative.

In conclusion, in guinea pigs of the same age, axial length was not correlated with body weight. Also, Baseline body weight had no impact on natural axial length growth or Lens-induced myopia. Further studies are required to determine the long-term effect of body weight on axial length elongation that on axial growth of each individual subject. Lens-induction has a negative has a negative effect on body weight gain in guinea pigs, and further studies are needed to clarify the biological significance of this effect.

## Nomenclature

BW = body weight [kg]

LIM = Lens Induced Myopia

AL = Axial length [mm]

BMI = Body Mass Index [kg/cm^2^]

## Data Availability

Data can be made available from the corresponding author on a reasonable request.
